# Nonlinear Origin of SSVEP Spectra—A Combined Experimental and Modeling Study

**DOI:** 10.3389/fncom.2016.00129

**Published:** 2016-12-27

**Authors:** Maciej Labecki, Rafal Kus, Alicja Brzozowska, Tadeusz Stacewicz, Basabdatta S. Bhattacharya, Piotr Suffczynski

**Affiliations:** ^1^Biomedical Physics Division, Faculty of Physics, Institute of Experimental Physics, University of WarsawWarsaw, Poland; ^2^Section of Optics, Faculty of Physics, Institute of Experimental Physics, University of WarsawWarsaw, Poland; ^3^School of Engineering, University of LincolnLincoln, UK

**Keywords:** steady state visual evoked potentials, SSVEP, flicker stimulation, computational modeling, harmonics, entrainment

## Abstract

Steady state visual evoked potentials (SSVEPs) are steady state oscillatory potentials elicited in the electroencephalogram (EEG) by flicker stimulation. The frequency of these responses maches the frequency of the stimulation and of its harmonics and subharmonics. In this study, we investigated the origin of the harmonic and subharmonic components of SSVEPs, which are not well understood. We applied both sine and square wave visual stimulation at 5 and 15 Hz to human subjects and analyzed the properties of the fundamental responses and harmonically related components. In order to interpret the results, we used the well-established neural mass model that consists of interacting populations of excitatory and inhibitory cortical neurons. In our study, this model provided a simple explanation for the origin of SSVEP spectra, and showed that their harmonic and subharmonic components are a natural consequence of the nonlinear properties of neuronal populations and the resonant properties of the modeled network. The model also predicted multiples of subharmonic responses, which were subsequently confirmed using experimental data.

## Introduction

Steady state visual evoked potentials (SSVEPs) are oscillatory brain responses to periodic light stimulation, and are observable in the electroencephalogram (EEG) (van der Tweel and VerduynLunel, 1965; Regan, 1966). These responses exhibit the same frequency as the frequency of the stimulation as well as its harmonics and subharmonics (Herrmann, 2001). Although, SSVEPs have widespread application in cognitive and clinical neuroscience (reviewed in Vialatte et al., 2010) and engineering (e.g., Müller-Putz et al., 2005; Bin et al., 2009; Guger et al., 2012), the mechanisms responsible for their generation are not yet fully understood. One feature still awaiting an explanation is related to the harmonic components in the response.

One stimulus commonly used to measure SSVEPs is a flickering stimulus, consisting of a periodic square wave with a 50% duty cycle and a Fourier spectrum containing only odd components (i.e., n•*f*_0_, n = 1, 3, 5,…, where *f*_0_ is the fundamental frequency). The SSVEP evoked by such a stimulus may contain both odd and even harmonics as well as subharmonic components. Harmonic frequencies also appear in the SSVEP elicited by sine wave stimulation in animals (Lopes da Silva et al., 1970a) and humans (van der Tweel and Spekreijse, 1969; Donker, 1975; Teng et al., 2011). These responses cannot be attributed to the harmonics of the stimulus, as a perfect sine wave does not contain higher harmonics.

It has been proposed that the first harmonic response (*n* = 2) of an SSVEP may be generated by a subset of cells in the visual system. Clynes et al. (1964) attributed the frequency doubling effect to “on” and “off” receptors in the retina. Similarly, McKeefry et al. (1996) showed that an achromatic stimulus, known to activate the magnocellular visual pathway, produced the maximum first harmonic component, and this result was diminished with a chromatic stimulus, known to activate the parvocellular visual pathway. The small parvocellular neurons (P cells), having more tonic (sustained) characteristics, generated larger responses for the onset of the stimulus than for the offset, and the response signal was dominated by the fundamental component. Conversely, the large magnocellular neurons (M cells), having more phasic (transient) responses, produced similar responses to the onset and offset of the stimulus and therefore contributed predominantly to the first harmonic component.

The association of first harmonic responses with magnocellular activity has been challenged on a number of grounds by Skottun and Skoyles (2007). They stated that although frequency doubling by individual receptors or neurons may contribute to the first harmonic component, this type of nonlinearity related to single cell properties probably cannot be the only factor affecting the characteristics of the SSVEP spectrum, e.g., the subharmonic and higher harmonic components. In general, it has been commonly assumed that harmonic and subharmonic frequencies are generated by nonlinearities of the visual system (Kelly, 1966; van der Tweel and Spekreijse, 1969; Lopes da Silva et al., 1970a; Regan and Regan, 1988; Vialatte et al., 2010; Roberts and Robinson, 2012; Norcia et al., 2015). These nonlinearities could be related to retinal, subcortical or cortical properties but their exact sources have never been explicitly shown.

In order to further clarify the mechanisms of the generation of the spectral components of SSVEPs, we analyze EEG recordings of SSVEP responses to square and sine wave stimulation at two different frequencies. The stimulation frequencies were lower and higher than frequencies within the alpha range (7–13 Hz)—at 5 and 15 Hz. We interpret the results using the computational neural mass model by Lopes da Silva et al. (1974), which takes into account the nonlinear properties of populations of cortical networks. Due to its simplicity, this model is considered as a basic model of brain rhythmicity, with output corresponding to EEG signals, and therefore it is well suited for our purpose.

It should be noted that Roberts and Robinson (2012) developed a corticothalamic neural field model consisting of four neuronal populations, which was able to reproduce many phenomena related to experimentally observed driven brain responses and predict new ones. Our aim is to show that an even simpler model of only two interacting populations can reproduce key features of SSVEP spectra and provide an easily understandable explanation of their origin. Our model generates testable predictions, which are subsequently validated using experimental data.

## Methods

### Experimental data

#### Subject and data collection

Ten healthy volunteers participated in this study (5 males and 5 females, mean age 24 years, range 21–29 years). The EEG signals and photodiode trigger signals were collected using the TMSiPorti 7 amplifier and modified 10–20 EEG cap (Easycap EC20). EEG signals were recorded with a linked ears reference (A1, A2) with a sampling frequency of 512 Hz, and were filtered offline with a high-pass third order Butterworth filter with a cut off frequency of 1 Hz to remove the DC component, and with a band stop 50 Hz first order Butterworth filter to remove line noise.

#### Visual stimulation

Subjects were seated on a comfortable chair, in a dim room. Visual stimulation was delivered using a custom-made SSVEP stimulator constructed for this experiment, placed 60 ± 20 cm in front of the subject. The stimulator consisted of an arbitrary wave shape generator and a lighting panel, which was backlighted by a diode. The evenly illuminated lighting surface was a 10 × 10 cm square. The stimulator enabled a sinusoidal stimulus wave shape with high accuracy (Total Harmonics Distortion, THD <2%) and a square stimulus wave shape with up to its 10 harmonics to be generated.

#### Experimental paradigm

The stimulation frequencies were chosen based on the fact that the strongest amplitude response of SSVEPs has been observed in stimulus frequency ranges of 5–10 Hz and 10–25 Hz by Regan (1975) and in the range of 5–25 Hz with a peak at around 15 Hz by Wang et al. (2006) and Pastor et al. (2003). After considering these reports, we decided to use the lower half of the reported range, as these lower stimulation frequencies might potentially generate a higher number of harmonics. Additionally, we avoided the alpha range (7–13 Hz) of stimulation frequencies in order to not interfere with strong spontaneous activity in this range. These two conditions led to the two stimulation frequencies of 5 and 15 Hz being selected.

Subjects were presented with two kinds of stimuli—sine wave and square wave. The choice of stimuli was not influenced by the neural mass model we use in this study, as the experimental paradigm was established prior to modeling considerations. The amplitudes of the light intensity of the sine and square wave stimuli were equal, hence the energy delivered to the system (the integral over light intensity) was the same for both types of stimuli. A single trial consisted of a 5 s resting period and 5 s of stimulation. There were 50 trials for each wave shape and frequency, for each subject. Trials for each stimuli type and frequency were delivered in blocks.

#### Data analysis

The 10 s EEG epochs corresponding to the experimental trials were extracted according to the photodiode trigger signal. Each epoch included a 5 s resting signal (prior to stimulation onset) and a 5 s response to the stimulus. For the purpose of the subharmonic response analysis (**Figure 5**), we also distinguished 1 s signals after stimulation onset and divided the resting signal into 1 s epochs. For each stimulation frequency and wave shape, the EEG signals were first averaged across trials, multiplied by the Hann window and finally their amplitude spectra were computed with Fast Fourier Transform (FFT), using the *fft* procedure in Matlab. Power spectra were calculated by first squaring and next log10 transforming the amplitude spectra. In general, such power estimates are not accurate as the power spectrum FFT estimator at a given frequency has a variance equal to the square of its expectation value at that frequency (Press et al., 1992). Typically, the power spectra of signal segments or of multiple realizations are averaged in order to reduce the variance (Manolakis et al., 2000). Here we followed an alternative approach and we first averaged phase-locked signals in order to increase the signal-to-noise ratio and then computed the power spectrum of the averaged signal. In this way, we reduced the variance of the final power estimate while preserving original frequency resolution. We used two different ways to show the spectral content of the SSVEPs. Dominant frequencies are more evident on the amplitude spectra, while higher harmonics are better visible on the power spectra in dB scale. The experimental results in **Figures 2, 3** are presented using data from the O2 EEG channel. To study subharmonic frequencies (**Figure 5**), data from electrodes P3, P4, and P8 were used, as these subharmonic responses were hardly detectable in the occipital O1 and O2 channels.

#### Statistical analysis

To assess the statistical significance of the harmonic and subharmonic components, we compared the spectra of EEG signals during stimulation periods and rest periods averaged for each subject and we tested the significance of the hypothesis that the median of differences between spectral power in these two conditions was zero. The significance was calculated using the one-sided Wilcoxon signed rank test. When analyzing the harmonic component of the SSVEP response, we additionally applied False Discovery Rate (FDR) correction for multiple comparisons (Benjamini and Yekutieli, 2001). The maximum FDR level (*q*-value) was set to 5%.

#### Ethics statement

All experimental protocols were approved by the Research Ethics Committee at the University of Social Sciences and Humanities in Warsaw, Poland. All methodological procedures were carried out in accordance with the approved guidelines. All subjects declared an absence of neurological and mental illnesses, and were screened for photosensitive epilepsy using the standard clinical EEG test. Informed, written consent was obtained from all subjects.

### Computational model

**Figure 1:**
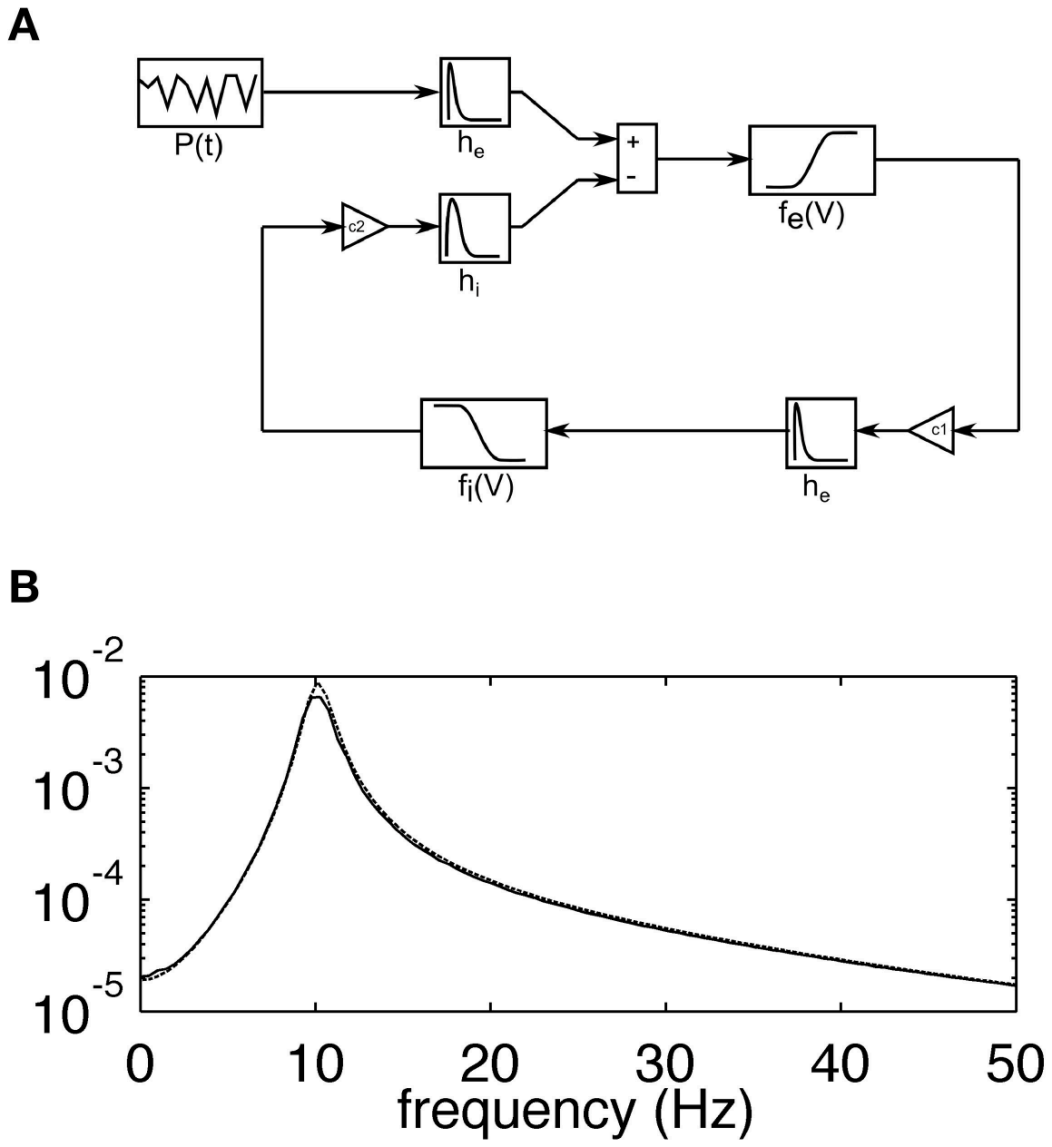
**(A)** Schematic diagram of the lumped alpha rhythm model. The main population of neurons is represented by impulse responses *he* and *hi* simulating excitatory and inhibitory postsynaptic potentials, respectively, and the sigmoidal function *f*_*e*_(*V*), which relates the average membrane potential to the firing rate of the population. The inhibitory neurons are represented by the impulse response *he* and the sigmoidal function *f*_*i*_(*V*). The coupling constants *C*_1_and *C*_2_ represent the average numbers of connections between respective cell types. The main population receives excitatory external input *P*(*t*) corresponding to sensory stimulation. **(B)** The transfer function of the model computed analytically using linear approximation (broken line; see (Suffczynski, 2000) for details of linear model analysis) and as a ratio between the power spectra of the simulated output and input signals (solid line). The y-axis is dimensionless. The almost perfect overlap of solid and broken lines and the absence of harmonics suggest that spontaneous alpha activity is generated in the linear model's regime.

The model used in this study corresponds to the lumped alpha rhythm model initially proposed by Lopes da Silva et al. (1974). It consists of two interacting heterogeneous populations of neurons. Excitatory cells of the main population project to the interneurons through excitatory AMPA synaptic connections while the latter population feeds back to the main cells with fast GABAA receptor mediated inhibitory postsynaptic potentials. The strength of interactions between the two populations is regulated by the constants *C*_1_ and *C*_2_, which describe the coupling from excitatory to inhibitory and from inhibitory to excitatory populations, respectively. A schematic diagram of the model is shown in Figure 1A, while its frequency characteristic, showing selectivity in the alpha range at around 10 Hz, is shown in Figure 1B. Each population is described by the time courses of postsynaptic potentials and a nonlinear sigmoidal transfer function, which describes the conversion between the mean membrane potential of a neuronal population and the firing rate of this population (number of pulses per second—pps). Synaptic responses were modeled with double exponential functions of the form:

$$\begin{array}{l} h_{syn}\left(t\right)=\;A_{syn}\left[\text{exp}\left(-a_{1syn}t\right)-\text{exp}\left(-a_{2syn}t\right)\right],\;a_{2syn}\\ \;>\;a_{1syn},\;syn=\left\{AMPA,\;GABAA\right\} \end{array}$$

Where, *a*_1*syn*_, *a*_2*syn*_ are synaptic decay and rise time constants, respectively, and *A*_*syn*_ is the amplitude of the synaptic response. Static nonlinear conversions from the mean membrane potential to firing rates in neuronal excitatory (e) and inhibitory (i) populations are of the sigmoidal form:

$$f_{k}\left(V_{k}\right)=\;G/(1+\text{exp}\left[(V_{k}-\theta )/\sigma \right]),\;k=\{e,\;i\}$$

Where *G* corresponds to amplitude, while θ and σ correspond to the threshold and slope, respectively. At the threshold value of the membrane potential, the firing rate of the population reaches half its maximal firing rate, while σ corresponds to the degree of population heterogeneity. The time evolution of the membrane potential *V*(*t*) resulting from the action potential sequence *Q*(*t*) arriving at a single synaptic connection described by the impulse response function *h*_*syn*_(*t*) has the general convolution form:

$$V\left(t\right)=\;\underset{-\infty }{\int^{t}}h_{syn}\left(t-\tau \right)Q\left(\tau \right)d\tau$$

The mean membrane potential of the excitatory population is modeled by convolving the incoming action potential density from the excitatory input (*P*) and the inhibitory population (*f*_*i*_) with the respective synaptic response functions. The mean membrane potential of the inhibitory population is modeled by convolving the incoming action potential density from the excitatory population (*f*_*e*_) with the respective synaptic response function.

All model parameters (13 in total) are the same as in the original publication (Lopes da Silva et al., 1974). Model parameters concerning synaptic responses and sigmoidal transformations were based on single cell measurements and were translated into neural mass model parameters as explained in Lopes da Silva et al. (1974). Additionally, the value of the external input of Gaussian white noise with mean 550 pps and standard deviation 10 pps was estimated based on the rate of spontaneous discharge in the optic tract. As an extension of the original model, we included the periodic external input, which corresponds to periodic visual stimulation. It is modeled as a sine or square wave with a mean of zero and amplitude of 120 pps. Periodic stimulation is linearly added to the input of Gaussian white noise and is fed to the main excitatory population through the AMPA synaptic impulse response function. Visual stimulation amplitude is the only parameter that has been added to the original model. Its value, 120 pps, was chosen by us in order to obtain results best matching the experimental data. Smaller/larger stimulation amplitudes led to smaller/larger amplitudes of SSVEP spectral peaks with respect to amplitudes of background activity.

Model output is a sum of postsynaptic potentials in the main cell population. The units of model output correspond to intracellular voltage and are in the millivolt (mV) range, while the units of typical EEG signals are in the order of microvolts (μV). This difference is due to the large difference between transmembrane and extracellular resistivity (neglecting membrane capacitive effects). Nevertheless, we assume that the dynamics of model output (e.g., its dominant frequency and harmonic components) correspond to experimentally observed local field potentials and EEG signals. The model was implemented using the Simulink toolbox in Matlab. Simulations were run using the ode3 (Bogacki-Shampine) integration method with a fixed time step of 2 ms, resulting in a sampling rate for simulated signals of 500 Hz.

#### Data analysis of simulated signals

All simulated signals were analyzed after initial transients died out. Spectra were computed in the same way as those of the experimental data. Amplitude spectra were obtained with FFT using the *fft* procedure in Matlab. Power spectra were computed by squaring and log10 transforming the amplitude spectra. For spectral analyses, single trials of *N* = 4096 data points were used. The plots in **Figure 4** were produced by running the model for a range of stimulation frequencies from 0.1 to 50 Hz with 0.1 Hz resolution and then computing the power spectrum for each run in the way described above. In the simulations in **Figure 4**, the noise component was removed from the input to improve the clarity of the plots, as with noise present, the patterns of harmonically related components were unchanged but more blurred. Colormaps were produced by mapping the spectral power values into colors (using the *pcolor* procedure in Matlab).

## Results

### Experimental and simulated SSVEP spectra

**Figure 2:**
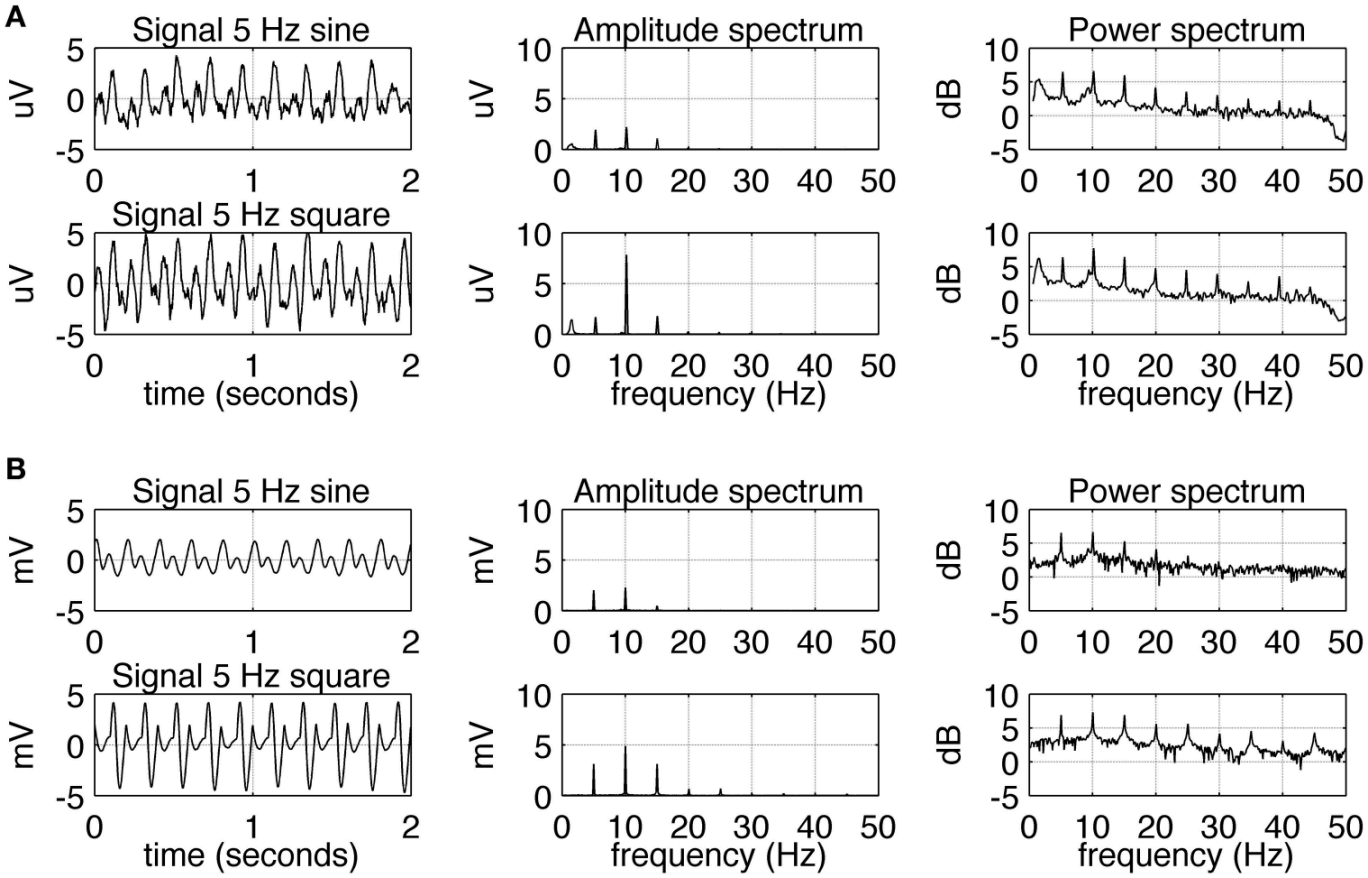
**Comparison of EEG recordings (A)** and modeling **(B)** results for 5 Hz stimulation. In each row, signals together with their amplitude and power spectra are shown. In part **(A)**, in order to increase the signal to noise ratio, the average spectra and signals for all 10 subjects are shown. A comparison of the first and second row shows that square wave stimulation evokes a stronger first harmonic (at 10 Hz) than sine wave stimulation does. All SSVEP peaks (fundamental components and harmonics) were statistically significant. In part **(B)**, the overall shape of the signals and spectra can be seen to be in accordance with the real data.

**Figure 3:**
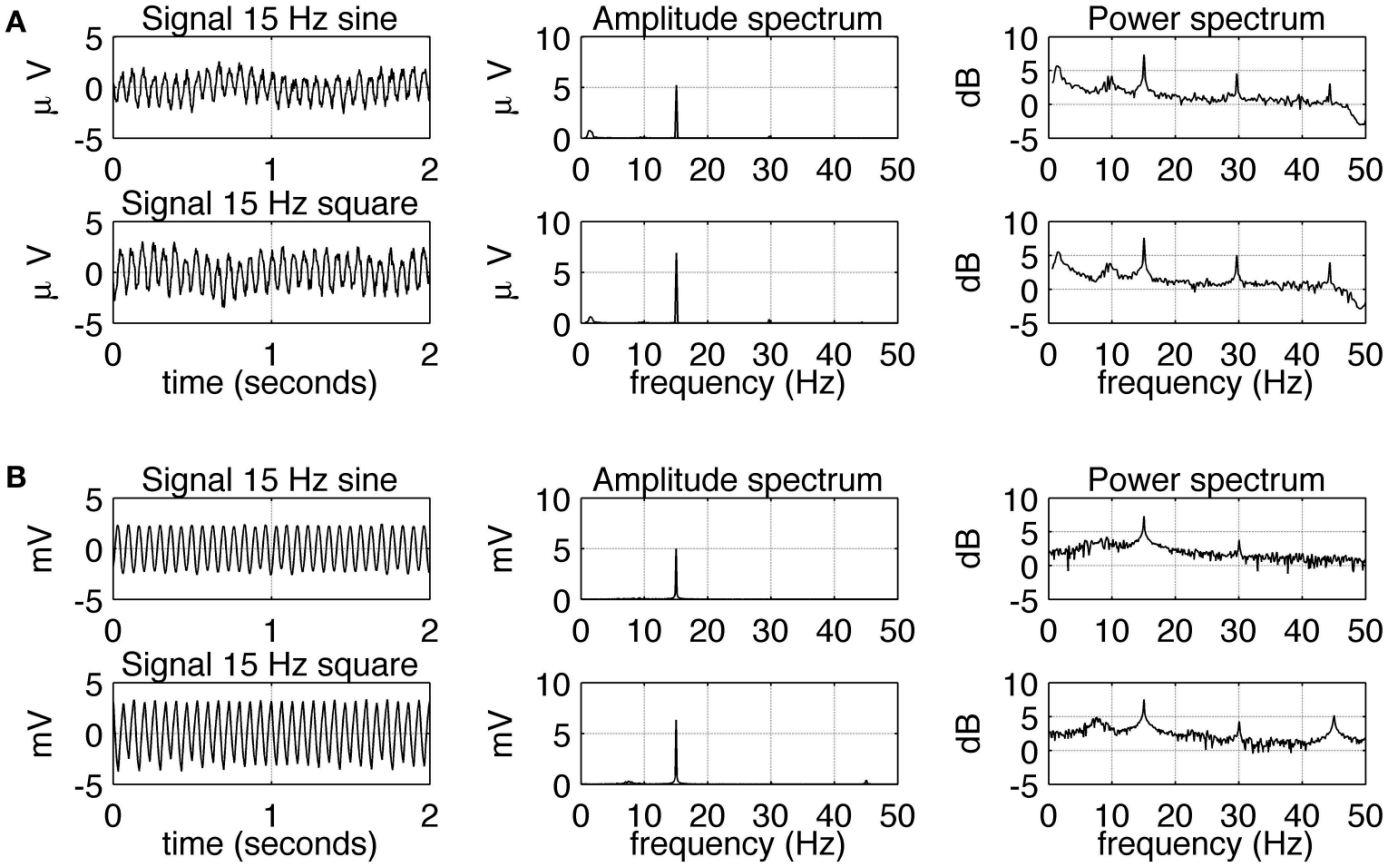
**Comparison of EEG recordings (A)** and modeling **(B)** results for 15 Hz stimulation. In each row, signals together with their amplitude and power spectra are shown. In part **(A)**, in order to increase the signal to noise ratio, average spectra and signals for all 10 subjects are shown. It can be seen that sine and square wave stimulation evoke similar responses, with the strongest response at the fundamental frequency of 15 Hz. All SSVEP peaks (fundamental components and harmonics) were statistically significant. In part **(B)**, the overall shape of the signals and spectra can be seen to be in accordance with the real data, but there is an absence of slow fluctuation of the envelope of the signal.

The experimental SSVEPs and their spectra recorded during sine and square wave stimulation at 5 Hz are shown in Figure 2A. It can be seen that both waveforms induce response at the fundamental and higher harmonic frequencies. The response at the first harmonic (*n* = 2) is larger than the response at the driving frequency (*n* = 1). This effect is more pronounced for square wave stimulation as the response at 10 Hz clearly dominates the amplitude spectrum (Figure 2A, middle column, bottom), but it is also present for sine wave stimulation (Figure 2A, middle column, top). The experimental SSVEPs and their spectra for 15 Hz stimulation are shown in Figure 3A. The responses at the driving frequency (*n* = 1) clearly dominate the amplitude spectra for both sine and square wave stimulation (Figure 3A, middle column, top and bottom). Higher harmonics (*n* = 2 and 3) are also present but since they are of smaller amplitude, they can only be seen on the logarithmic power spectra (Figure 3A, right column, top and bottom). It can be also noticed that the spectral amplitudes of the responses to sine and square wave stimulation are comparable (Figure 3A, middle column, top vs. bottom), contrary to 5 Hz stimulation, where the response to square wave stimulation is larger than the response to sine wave input (Figure 2A, middle column, top vs. bottom). All SSVEP peaks (fundamental components and harmonics) in Figures 2, 3 were statistically significant after FDR correction, with *p*-values lower than 0.0185 and 0.001 for 5 and 15 Hz stimulation, respectively.

Simulated SSVEPs and spectra for 5 and 15 Hz stimulation are shown in Figures 2B, 3B, respectively. A close resemblance between real SSVEP recordings and simulated SSVEPs, including similarity in signal shapes as well as in positions and magnitudes of spectral peaks, can be seen. The main difference is the slow modulation of the amplitude of experimental signals compared to the signals generated by the model, which look more stable. The modulation is caused by the low frequency components of the EEG signal that were not entirely filtered out by the low pass filter.

### Neural mechanisms generating SSVEP harmonics

**Figure 4:**
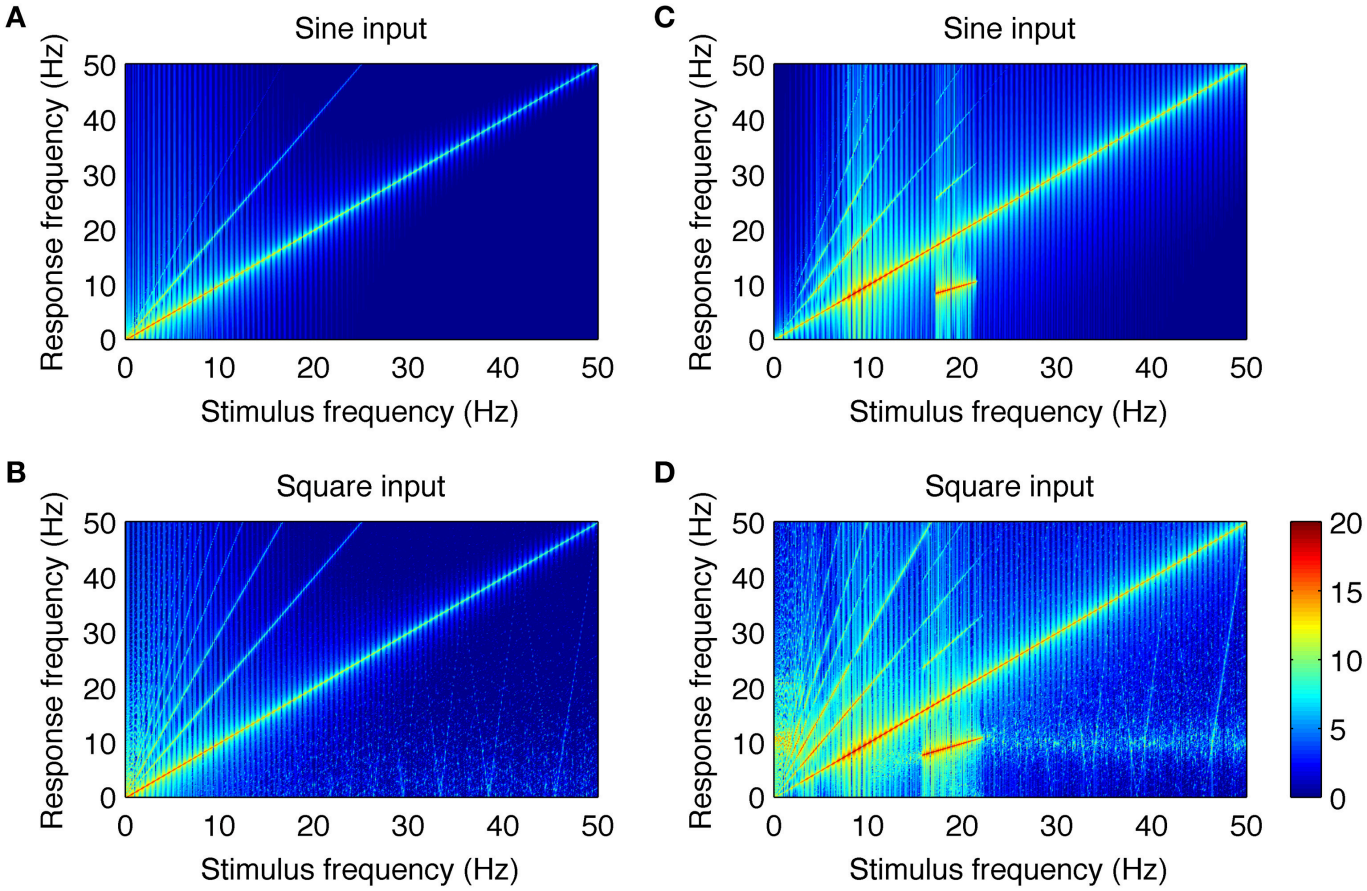
**Analysis of the neural mechanisms generating SSVEP harmonics in the model**. In each plot, the response frequency (y-axis) as a function of the stimulus frequency (x-axis) is shown. Color represents the spectral power of the response calculated as squared and log10 transformed amplitude spectrum. The color bar showing the logarithmic power scale in dB is shown at the bottom right. Parts **(A,B)** show responses to sine and square wave stimulation in the model with coupling constant *C*_2_ = 0 and with the mean membrane potential of the inhibitory population taken as the model output. Under this condition, the signal passes through only one nonlinearity. It can be noticed that there are new spectral components that were not present in the input signals. They occur at integer multiples of the stimulus frequency and do not contain subharmonic frequencies. Parts **(C,D)** show responses to sine and square wave stimulation in the intact circuit, where the signal passes through both nonlinear transformations. More complex response patterns can be observed, including additional higher harmonic components and subharmonic responses for stimulation frequencies of 17–21.5 Hz **(C)** and 15–22 Hz **(D)**. Overall, this figure shows that experimentally observed SSVEP spectra can be well explained by two interacting populations with nonlinear characteristics.

The close resemblance between real and simulated SSVEPs and spectra suggests that the computational model can be used to investigate the origin of whole number relations observed between stimulus and response frequency. As the only nonlinear elements in the model are the two static nonlinear transformations *f*_*e*_(*V*), *f*_*i*_(*V*), we looked at their roles in the generation of harmonic components of SSVEPs. To this end, we applied a systematic model analysis and stimulated the model with sine and square wave periodic input for a range of stimulation frequencies as described in the Methods section. Firstly, we performed the analysis in an “open loop” condition, where the input signal passed through only one nonlinearity. This was achieved by setting the coupling constant *C*_2_ to zero (see Figure 1A) and analyzing the mean membrane potential of the inhibitory population, i.e., the signal after transformation *f*_*e*_(*V*) but just before transformation *f*_*i*_(*V*). Secondly, we analyzed the intact circuit of two interacting populations and the mean membrane potential of the excitatory population, i.e., the signal after both transformations *f*_*e*_(*V*), *f*_*i*_(*V*). All other blocks in the model are linear and may influence the amplitude and phase of the input frequencies, but cannot generate new spectral components. The results are summarized in Figure 4.

Figures 4A,B show the responses to sine and square wave inputs, respectively, after single nonlinear transformation *f*_*e*_(*V*). These figures show new spectral components that were not present in the input signals. This is most evident for sine wave stimulation (Figure 4A) as the response contains second and third harmonics of the stimulus frequency. Similarly, the response to square wave input (Figure 4B) contains even and odd harmonics, although only odd components are present in the input. Although, new spectral components can be observed after single nonlinear transformation, these components occur at exact integer multiples of the stimulus frequency and do not contain subharmonic responses. Responses to sine and square wave inputs in the full model, containing two nonlinear transformations, are presented in Figures 4C,D. It can be noticed that additional subharmonic frequency components appear in the response to both sine and square wave inputs. These subharmonic responses are observed for stimulation frequencies in the range of 17–21.5 Hz (Figure 4C) and 15–22 Hz (Figure 4D). The first subharmonic response (*n* = 1/2) corresponds to the alpha frequency range at around 8–11 Hz, while the higher subharmonic responses (*n* = 3/2 and 5/2) visible in Figures 4C,D correspond to its multiples. A comparison of Figures 4C,D shows that after the two nonlinear transformations, the response patterns become comparable for both sine and square wave inputs. The main difference is in the small-power, higher harmonic components (*n* > 4) and weak alpha frequency response present for the square wave above the 15–22 Hz range but absent for the sine wave.

The higher subharmonic components (*n* = 3/2 and 5/2) present in Figures 4C,D have not been reported in experimental data so far, and thus may be considered as novel model predictions. They were in turn examined using SSVEPs recorded during 15 Hz square wave stimulation.

### Analysis of subharmonic responses

**Figure 5:**
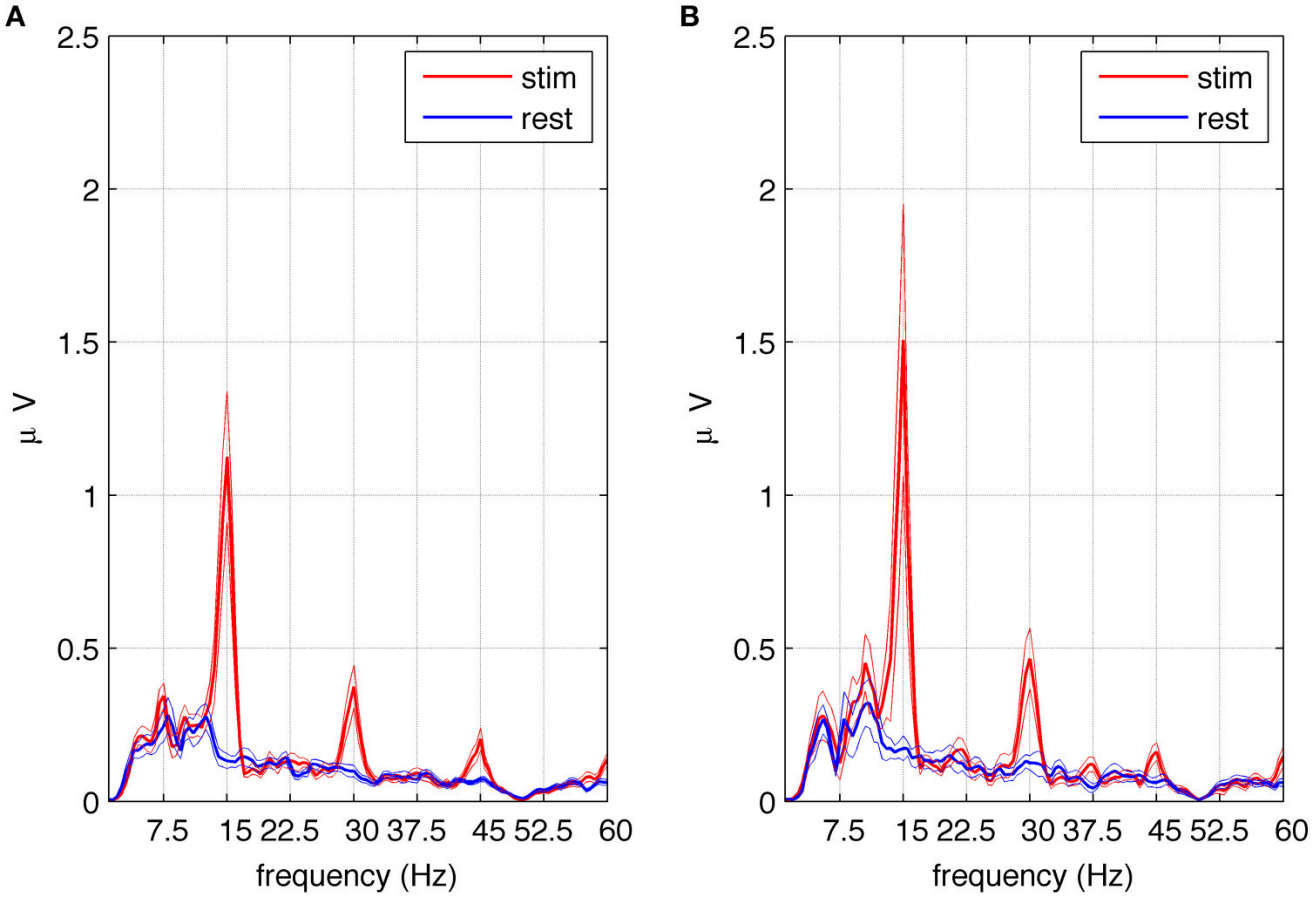
**Amplitude spectra of SSVEPs in the selected group of eight subjects exhibiting subharmonic responses to square wave stimulation at 15 Hz**. Part **(A)** shows the average spectra of signals from electrodes P4 and P8 during stimulation (red) and rest periods (blue) together with the standard error of the mean. Multiples of the fundamental frequency (*n* = 1, 2, 3) can be seen. The first subharmonic response (*n* = 1/2) visible at 7.5 Hz is statistically significant as well as the fundamental component and harmonics. Part **(B)** shows the average spectra of signals from the referential montage of electrodes P8–P3 during stimulation (red) and rest periods (blue) together with the standard error of the mean. Multiples of the fundamental frequency (*n* = 1, 2, 3) can be seen together with the third subharmonic response visible at 37.5 Hz. The fundamental component, harmonics and subharmonics (*n* = 5/2) are statistically significant.

A response at the first subharmonic frequency of 7.5 Hz (*n* = 1/2) was observed in eight out of 10 subjects during the first second after stimulation onset. All subsequent analyses described in this section were performed on this selected group of subjects. The highest magnitude of first subharmonic response was present at the P4 or P8 electrode, depending on the subject. Therefore, for the purpose of further analysis we averaged spectral amplitudes from these two electrodes. The average amplitude spectrum across eight subjects and the two selected electrodes is shown in Figure 5A. Harmonic responses (*n* = 1, 2, 3) as well as the first subharmonic (*n* = 1/2) are visible. All harmonic responses were statistically significant, as reported earlier for all 10 subjects.

To assess the significance of the first subharmonic response, 1 s stimulation epochs and 1 s resting periods were averaged, and the spectral amplitudes were compared in these two conditions (see Section Data Analysis). An increase in EEG amplitude at the first subharmonic frequency (*n* = 1/2) was found, and determined to be significant at *p* < 0.019. To test the significance of higher subharmonic frequencies, i.e., 22.5 and 37.5 Hz, we used a referential montage of electrodes in order to remove common sources of EEG activity present in both resting and stimulated epochs. We decided to use the classic bipolar montage P4-P8 and its analog P8-P3. The second subharmonic at 22.5 Hz was observed but did not reach statistical significance, while the third subharmonic gave critical *p* values at the statistical significance threshold for the classic bipolar montage (*p* < 0.055) and below the threshold for the other montage (*p* < 0.020). The average amplitude spectrum across eight subjects for the referential P8-P3 montage is shown in Figure 5B, where the peak at 37.5 Hz is significantly higher during stimulation (red line) than during resting periods (blue line).

## Discussion

The aim of this work is to examine harmonic and subharmonic components of real SSVEPs and SSVEPs from a simple computational model, in order to provide additional insight into their generation. First we showed that both sine wave stimulus (which contains no harmonics) and square wave stimulus (which contains only odd multiples of fundamental frequency) induced both even and odd harmonics in the power spectra of the recorded EEG signals (Figures 2, 3). This is in agreement with results from Teng et al. (2011), and suggests that the appearance of harmonics in an SSVEP spectrum cannot be simply explained by their presence in the input. Instead, they may arise due to the nonlinear transformation of the input by the visual system. Such a possibility was suggested in early SSVEP studies (Kelly, 1966; van der Tweel and Spekreijse, 1969; Lopes da Silva et al., 1970a; Regan and Regan, 1988), and the origin of various forms of nonlinearities i.e., saturation, nonlinear oscillations responsible for the generation of subharmonics, and essential nonlinearities, was attributed to various stages of processing in the retina-cortex system. E.g., Clynes et al. (1964) noted that cortical evoked potentials triggered by increasing and decreasing rates of light intensity had the same polarity, thus doubling the stimulation frequency. They related this to various “on” and “off” receptors in the retina. It was subsequently observed by Lopes da Silva et al. (1970a) that the essential nonlinearities corresponding to the rectification of “on” and “off” responses to light stimulation were mainly dominant in the lateral geniculate nucleus, which exhibited marked frequency doubling. In line with these studies, McKeefry et al. (1996) observed that response to chromatic stimulation was dominated by the fundamental component, while achromatic stimulation additionally triggered a second harmonic component, which was attributed to magnocellular neurons with transient characteristics. Saturation-related nonlinearities were also observed at large modulation depths (the ratio of the modulation amplitude and the carrier amplitude) of 40–80%, but were shown not to be the primary factor affecting the appearance of harmonics at these depths (Lopes da Silva et al., 1970a). Another type of nonlinearity responsible for the generation of subharmonics was identified at the cortical level, where subharmonics were exclusively found (Lopes da Silva et al., 1970a).

The early SSVEP studies used mathematical, descriptive models to analyze characteristics of the visual system under periodic light stimulation. These models of both nonlinear (e.g., Kelly, 1966) and linear (e.g., Lopes da Silva et al., 1970b) type showed that static transfer functions can accurately describe both amplitude and phase characteristics of SSVEPs, but they did not determine the physiological mechanisms underlying the observed responses. Recently, Roberts and Robinson (2012) developed a physiologically based neural field model of the thalamocortical system that reproduced many features of nonlinear cortical responses to periodic light stimulation. An elegant and extensive analysis of the model's properties revealed a number of intriguing phenomena to be considered in future experiments, including chaotic behavior. The results concerning the spectral properties of SSVEPs are similar to our results (e.g., the existence of harmonics and subharmonics was demonstrated). Nevertheless, the work by Roberts and Robinson (2012) focuses mainly on studying the mathematical properties of the model, while our study aims to provide a model-based interpretation of the observed SSVEPs regarding, e.g., differences between sine- and square-evoked signals and the relative height of peaks in their spectra. Our results indicate that even a simpler, well-established, cortical neural mass model is capable of explaining a number of properties of driven EEG signals.

Comparing the SSVEP responses to 5 and 15 Hz stimulation reveals two main features. The first feature is that the amplitude spectrum for 5 Hz stimulation contains strong responses—the fundamental response at 5 Hz as well as strong harmonic responses at 10 and 15 Hz (Figure 2A), while the amplitude spectrum for 15 Hz stimulation contains mainly the fundamental response at 15 Hz (Figure 3A). In both cases, harmonic responses above 15 Hz are present as can be seen on the corresponding power spectra in the logarithmic scale, but these higher frequency components are significantly attenuated. The attenuation and hence the observed difference between the number of strong harmonics at 5 and 15 Hz stimulation can be explained using the computational model by considering the properties of the transfer function of the modeled network. As can be seen in Figure 1B, the network has a dominant peak at around 10 Hz, and the magnitude of the response decreases sharply for frequencies further away from the peak frequency. This may explain why the higher harmonics of 15 Hz stimulation, e.g., 30 and 45 Hz, are not visible on the amplitude spectra (Figures 3A,B, middle panel).

The second notable feature of the SSVEP responses to 5 and 15 Hz stimulation is that the difference between responses to sine and square wave stimulation is only present for 5 Hz stimulation (Figure 2A) and is absent for 15 Hz stimulation (Figure 3A). This difference is manifested by the first harmonic (10 Hz) being much larger than the fundamental response (5 Hz) for square wave stimulation compared to sine wave stimulation, where fundamental and first harmonic responses are comparable (Figure 2A). In the computational model, the increase of the first harmonic (10 Hz) for 5 Hz stimulation is also present and can be attributed to the phenomenon of entrainment. In general, entrainment refers to synchronization of two or more independent oscillators with differing natural frequencies, due to their coupling. In our study, entrainment by the first harmonic of the stimulus occurs because its frequency (10 Hz) is close to the natural resonant frequency of the model (Figure 1B). Entrainment of alpha activity by the first harmonic (~5 Hz light flashes; Miranda de Sá and Infantosi, 2005), and by the first harmonic (~5 Hz light flashes) and second harmonic (~3.5 Hz light flashes; Gebber et al., 1999) has been reported before, but in these studies the subjects were stimulated by periodic flashes, which contained odd multiples of stimulation frequency in their spectra. In our study, entrainment of alpha rhythm was also present for pure sine wave stimulus at 5 Hz, supporting a hypothesis of a nonlinear origin of this phenomenon. Alternatively to an entrainment hypothesis, the first harmonic response (10 Hz) for 5 Hz stimulation could arise due to rectification of “on” and “off” responses as e.g., suggested by Clynes et al. (1964) and termed “essential nonlinearities” (Kamp et al., 1960; van der Tweel, 1961; Lopes da Silva et al., 1970a). Our results do not reject the existence of such essential nonlinearities but do not confirm them either. In the computational model, the frequency doubling phenomenon is not present as the “on” and “off” responses have different waveforms and opposite polarities. This shows that the presence of essential nonlinearities is not necessary to explain the observed results.

Another phenomenon related to the nonlinear properties of the modeled network is associated with subharmonic frequencies. These subharmonic responses are observed in the computational model for sine wave stimulation in the range of 17–21.5 Hz (Figure 4C) and for square wave stimulation in the range of 15–22 Hz (Figure 4D). The first subharmonic response at 1/2 *f*_0_, with *f*_0_ being the fundamental frequency, is observed at the resonant network frequencies (i.e., the 7.5–11 Hz range). Higher harmonics of the subharmonic response (3/2 and 5/2 *f*_0_) are also present in simulated signals. The appearance of these subharmonic responses in the model is related to interactions between the two neuronal populations with nonlinear characteristics. This can be inferred by comparing Figures 4A,B with Figures 4C,D. A single nonlinear transformation of the input may produce only harmonic responses that are exact multiples of the stimulus frequency (Figures 4A,B). Two nonlinear transformations arranged with negative feedback are sufficient to generate a resonant circuit and subharmonic responses (Figures 4C,D). The first subharmonic at the alpha frequency range is related to the entrainment of the resonant frequency by the stimulus with double alpha frequency. The subharmonic response does not have a purely sinusoidal shape, and this is manifested by its discrete spectrum with peaks at n/2 *f*_0_, *n* = 1, 3, 5.…

First subharmonic responses have been observed experimentally. Lopes da Silva et al. (1970a) showed that a subharmonic at 1/2 *f*_0_ was present mainly for the stimulation range of 32–38 Hz at the end of the typical 6 s response. In a study by Herrmann (2001), subharmonic responses in the alpha frequency range (9–13 Hz) were reported for stimulation frequencies in the range of 17–25 Hz. Similarly, it was argued that stimulation in the 24–27 Hz range might cause alpha range synchronization through subharmonics of the stimulus (Angelini et al., 2004).

Although multiples of the first subharmonic response were predicted by Roberts and Robinson (2012) and by the model in the present study, they have never been reported in experimental data, to the best of our knowledge. After thorough examination of the recorded SSVEPs, we found evidence to validate these predictions. In Figures 5A,B, the amplitude spectra for 15 Hz square wave stimulation contain peaks at fundamental (15 Hz) and harmonic (30 and 45 Hz) frequencies. Depending on electrode position and montage, subharmonics at 7.5 (Figure 5A) and at 37.5 Hz (Figure 5B) are also visible. Another predicted subharmonic at 22.5 Hz was visible as well at some electrodes, but it never reached a statistical significance, probably because the peak was masked by broad-band beta activity (15–30 Hz). It should be emphasized that subharmonic frequencies were detected only in eight out of 10 subjects, mainly at the beginning of the stimulation, and tended to occur in parietal (P) rather the occipital (O) electrodes.

While our computational model can reproduce many experimentally observable facts and can relate them to general nonlinear characteristics of neuronal populations, it is still only a simplified approximation of real brain networks and cannot account for the full spectrum of spontaneous SSVEP responses. E.g., a spontaneous SSVEP spectrum may contain distinct local peaks that correspond to various brain rhythms. The spontaneous activity generated by the model without stimulation has single dominant frequency in the alpha range (Figure 1B). Furthermore, harmonically related responses have been observed for stimulation frequencies up to 50 Hz (Figure 7 in Herrmann, 2001), but our model does not generate harmonic responses for stimulation frequencies above 25 Hz (Figures 4C,D) as these higher frequencies are strongly attenuated due to model band-pass frequency characteristics. This may be considered as a limitation of the model, and is a consequence of its simplicity.

In summary, a simple neural mass model was used to explain a number of features observed in SSVEP responses to visual stimulation. Early studies of cortical and subcortical responses to periodically modulated light attributed the origin of SSVEP spectral components to various forms of nonlinearities i.e., saturation, nonlinear oscillations responsible for the generation of subharmonics and essential nonlinearities. Our study showed that two cortical neuronal populations described by nonlinear sigmoidal characteristics might account for observed SSVEP spectra, despite none of the earlier suggested types of nonlinearities being explicitly present in the model (except saturation, but it was never reached during model operation). The model predicted some new SSVEP characteristics that were subsequently confirmed by the experimental data, and this increases our confidence in the value of the model. Thus, our combined experimental and modeling study may be considered a step forward toward full understanding of the physiological processes involved in generating harmonically related SSVEP responses.

## Author contributions

ML: experimental data acquisition, experimental data analysis, writing of the manuscript. RK: experimental data acquisition, experimental data analysis, conception of the work, revising. AB: experimental data acquisition, drafting. TS: experimental data acquisition, revising. BB: simulation data analysis, revising. PS: conception of the work, simulation data analysis, writing, editing and revising of the manuscript.

### Conflict of interest statement

The authors declare that the research was conducted in the absence of any commercial or financial relationships that could be construed as a potential conflict of interest.

## References

[B1] Angelini L. De Tommaso M. Guido M. Hu K. Ivanov P. Ch. Marinazzo D.. (2004). Steady-state visual evoked potentials and phase synchronization in migraine patients. Phys. Rev. Lett. 93:038103. 10.1103/PhysRevLett.93.038103 15323876

[B2] Benjamini Y. Yekutieli D. (2001). The control of the false discovery rate under dependancy. Ann. Statist. 29, 1165–1188. 10.1214/aos/1013699998

[B3] Bin G. Gao X. Yan Z. Hong B. Gao S. (2009). An online multi-channel SSVEP-based brain-computer interface using a canonical correlation analysis method. J. Neural Eng. 6:046002. 10.1088/1741-2560/6/4/046002 19494422

[B4] Clynes M. Kohn M. Lifshitz K. (1964). Dynamic and spatial behaviour of light evoked potentials: their modification under hypnosis and on-line correlation in relation to rhythmic components. Ann. N.Y. Acad. Sci. 112, 468–509. 10.1111/j.1749-6632.1964.tb26764.x 14188111

[B5] Donker D. N. (1975). Harmonic composition and topographic distribution of responses to sine wave modulated light (SML), their reproducibility and their interhemispheric relationship. Electroencephalogr. Clin. Neurophysiol. 39, 561–5574. 10.1016/0013-4694(75)90069-3 53136

[B6] Gebber G. L. Zhong S. Lewis C. Barman S. M. (1999). Human brain alpha rhythm: nonlinear oscillation or filtered noise? Brain Res. 818, 556–560. 10.1016/s0006-8993(98)01303-1 10082847

[B7] Guger C. Allison B. Z. Großwindhager B. Prückl R. Hintermüller C. (2012). How many people could use an SSVEP BCI? Front. Neurosci. 6:169. 10.3389/fnins.2012.00169 23181009 PMC3500831

[B8] Herrmann C. S. (2001). Human EEG responses to 1–100 Hz flicker: resonance phenomena in visual cortex and their potential correlation to cognitive phenomena. Exp. Brain Res. 137, 346–353. 10.1007/s002210100682 11355381

[B9] Kamp A. Sem-Jacobsen C. W. Storm Van Leeuwen W. van der Tweel L. H. (1960). Cortical responses to modulated light in the human subject. Acta Physiol. Scand. 48, 1–12. 10.1111/j.1748-1716.1960.tb01840.x 14404245

[B10] Kelly D. H. (1966). Frequency doubling in visual responses. J. Opt. Soc. Am. 56, 1628–1633. 10.1364/JOSA.56.001628

[B11] Lopes da Silva F. H. Hoeks A. Smits H. Zetterberg L. H. (1974). Model of brain rhythmic activity. The alpha-rhythm of the thalamus. Kybernetik 15, 27–37. 10.1007/BF00270757 4853232

[B12] Lopes da Silva F. H. Van Rotterdam A. Storm Van Leeuwen W. Tielen A. M. (1970a). Dynamic characteristics of visual evoked potentials in the dog. I. Cortical and subcortical potentials evoked by sine wave modulated light. Electroenceph. Clin. Neurophysiol. 29, 246–259. 10.1016/0013-4694(70)90137-9 4195647

[B13] Lopes da Silva F. H. van Rotterdam A. Storm van Leeuwen W. Tielen A. M. (1970b). Dynamic characteristics of visual evoked potentials in the dog. II. Beta frequency selectivity in evoked potentials and background activity. Electroencephalogr. Clin. Neurophysiol. 29, 260–268. 10.1016/0013-4694(70)90138-0 4195648

[B14] Manolakis D. G. Ingle V. K. Kogon S. M. (2000). Statistical and Adaptive Signal Processing: Spectral Estimation, Signal Modeling, Adaptive Filtering and Array Processing. New York, NY: The McGraw-Hill Companies, Inc.

[B15] McKeefry D. J. Russell M. H. Murray I. J. Kulikowski J. J. (1996). Amplitude and phase variations of harmonic components in human achromatic and chromatic visual evoked potentials. Vis. Neurosci. 13, 639–653. 10.1017/S0952523800008543 8870222

[B16] Miranda de Sá A. M. Infantosi A. F. (2005). Evaluating the entrainment of the alpha rhythm during stroboscopic flash stimulation by means of coherence analysis. Med. Eng. Phys. 27, 167–173. 10.1016/j.medengphy.2004.09.011 15642512

[B17] Müller-Putz G. R. Scherer R. Brauneis C. Pfurtscheller G. (2005). Steady-state visual evoked potential (SSVEP)-based communication: impact of harmonic frequency components. J. Neural Eng. 2, 123–130. 10.1088/1741-2560/2/4/008 16317236

[B18] Norcia A. M. Appelbaum L. G. Ales J. M. Cottereau B. R. Rossion B. (2015). The steady-state visual evoked potential in vision research: a review. J. Vis. 15:4. 10.1167/15.6.4 26024451 PMC4581566

[B19] Pastor M. A. Artieda J. Arbizu J. Valencia M. Masdeu J. C. (2003). Human cerebral activation during steady-state visual-evoked responses. J. Neurosci. 23, 11621–11627. 14684864 10.1523/JNEUROSCI.23-37-11621.2003 PMC6740964

[B20] Press W. H. Teukolsky S. A. Vetterling W. T. Flannery B. P. (1992). Numerical Recipes in C: The Art of Scientific Computing, 2nd Edn. New York, NY: Cambridge University Press.

[B21] Regan D. (1966). Some characteristics of average steady-state and transient responses evoked by modulated light. Electroencephalogr. Clin. Neurophysiol. 20, 238–248. 10.1016/0013-4694(66)90088-5 4160391

[B22] Regan D. (1975). Recent advances in electrical recording from the human brain. Nature 6, 401–407. 10.1038/253401a0 1089209

[B23] Regan M. P. Regan D. (1988). A frequency domain technique for characterizing nonlinearities in biological systems. J. Theor. Biol. 133, 293–317. 10.1016/S0022-5193(88)80323-0

[B24] Roberts J. A. Robinson P. A. (2012). Quantitative theory of driven nonlinear brain dynamics. Neuroimage 62, 1947–1955. 10.1016/j.neuroimage.2012.05.054 22652022

[B25] Skottun B. C. Skoyles J. R. (2007). Some remarks on the use of visually evoked potentials to measure magnocellular activity. Clin. Neurophysiol. 118, 1903–1905. 10.1016/j.clinph.2007.06.007 17644032

[B26] Suffczynski P (2000). Neural Dynamics Underlying Brain Thalamic Oscillations Investigated with Computational Models. doctoral thesis, University of Warsaw, Warsaw. Available online at: http://www.fuw.edu.pl/~suffa/phd.pdf

[B27] Teng F. Chen Y. Choong A. M. Gustafson S. Reichley C. Lawhead P.. (2011). Square or sine: finding a waveform with high success rate of eliciting SSVEP. Comput. Intell. Neurosci. 2011, 1–5. 10.1155/2011/364385 21941529 PMC3173954

[B28] van der Tweel L. H. (1961). Some problems in vision regarded with respect to linearity and frequency response. Ann. N.Y. Acad. Sci. 89, 829–856. 10.1111/j.1749-6632.1961.tb20181.x 13778838

[B29] van der Tweel L. H. Spekreijse H. (1969). Signal transport and rectification in the human evoked-response system. Ann. N.Y. Acad. Sci. 156, 678–695. 10.1111/j.1749-6632.1969.tb14007.x 5258014

[B30] van der Tweel L. H. VerduynLunel H. F. E. (1965). Human visual responses to sinusoidally modulated light. Eleetroenceph. Clin. Neurophysiol. 18, 587–598. 10.1016/0013-4694(65)90076-3 14296836

[B31] Vialatte F. B. Maurice M. Dauwels J. Cichocki A. (2010). Steady-state visually evoked potentials: focus on essential paradigms and future perspectives. Prog. Neurobiol. 90, 418–438. 10.1016/j.pneurobio.2009.11.005 19963032

[B32] Wang Y. Wang R. Gao X. Hong B. Gao S. (2006). A practical VEP-based brain-computer interface. IEEE Trans. Neural Syst. Rehabil. Eng. 14, 234–239. 10.1109/TNSRE.2006.875576 16792302

